# The Role of the Pediatric Dentist in the Multidisciplinary Management of the Cleft Lip Palate Patient

**DOI:** 10.3390/ijerph18189487

**Published:** 2021-09-08

**Authors:** Valeria Luzzi, Giulia Zumbo, Mariana Guaragna, Gabriele Di Carlo, Gaetano Ierardo, Gian Luca Sfasciotti, Maurizio Bossù, Iole Vozza, Antonella Polimeni

**Affiliations:** Department of Oral and Maxillo-Facial Sciences, Sapienza University of Rome, 00161 Rome, Italy; valeria.luzzi@uniroma1.it (V.L.); mariana.guaragna@uniroma1.it (M.G.); gabriele.dicarlo@uniroma1.it (G.D.C.); gaetano.ierardo@uniroma1.it (G.I.); gianluca.sfasciotti@uniroma1.it (G.L.S.); maurizio.bossu@uniroma1.it (M.B.); iole.vozza@uniroma1.it (I.V.); antonella.polimeni@uniroma1.it (A.P.)

**Keywords:** cleft lip palate, pediatric dentistry, multidisciplinary management, multidisciplinary team

## Abstract

The focus of this paper is the pediatric dental care of Cleft Lip and Palate (CLP) children and the role of the pediatric dentist in the CLP team. The management of children with cleft lip and palate presents many challenges and a multidisciplinary and prepared team is always required. Affected individuals present a multiplicity of problems: effective management involves a wide range of specialities. The value of a multidisciplinary team is widely known and mentioned in the literature, but very few papers focus on the role and the importance of the pediatric dentist. Therefore, the purpose of this article is to underline the role of the pediatric dentist as a member of the cleft lip and palate team which ranges from prenatal counseling, presurgical prevention and orthopedics, to post-treatment rehabilitation and restoration.

## 1. Introduction

Cleft lip and palate (CLP) are craniofacial dysmorphisms that fall within the anomalies of the developmental jaws as they are congenital malformations characterized by the arrested development of the homonymous regions of the maxillofacial district. Orofacial clefts occur due to failure of migration or fusion in the embryonic period of intrauterine life; craniofacial skeletal structures, hard and soft tissues of the oral cavity are particularly involved. The cause of cleft lip and/or palate (CLP) is thought to be multifactorial, namely through genetic or environmental factors [1,2].

In literature, recent studies express consensus about the prevalence at birth of CLP, which on average is around 1 over 500 live births in eastern countries and decreases to 1 over 1000 live births in western countries [3,4,5,6,7,8,9].

### 1.1. Cleft Lip and Palate-Related Orodental Issues in the Pediatric Age

Oral problems occurring in the pediatric age in subjects affected by cleft lip and palate are characterized by both dental anomalies and oral health problems. The latter are linked not only to oral anomalies, but also to the presence of cicatricial results from early maxillary orthopedics and surgery which interfere with adequate oral hygiene maneuvers and consequentially increase the risk of tooth decay and gingivitis [10,11]. The several orodental problems that might affect pediatric CLP patients are: anomalies of number (excess and defect), anomalies of shape, anomalies of volume, anomalies of seat, anomalies of structure, eruption anomalies, maxillary bones growth deficit, poor oral hygiene and related risk of gingivitis and dental caries [12,13].

Recent epidemiological studies aimed at evaluating the prevalence of pediatric dentistry problems associated with CLP. A very recent case-control study [14] was conducted on Columbian pediatric patients with non-syndromic CLP aged between 5 and 12 years. The sample consisted of 210 CLP subjects and 210 healthy subjects. Only dental anomalies affecting permanent teeth were considered. CLP patients presented a significantly greater risk (*p* < 0.0001) of developing agenesis of the maxillary lateral incisors, supernumerary teeth, microdontia of the maxillary lateral incisors, and rotation of the maxillary central incisor adjacent to the cleft. Most anomalies were located in the cleft area. In particular, the highest prevalence was found for the microdontia of the lateral maxillary incisors, followed by rotations of the central maxillary incisors, agenesis of the lateral maxillary incisors, and supernumerary teeth.

A Japanese longitudinal study from 2017 [13] studied a large sample of CLP patients in the period from 1970 to 2009 with the aim of collecting data on dental anomalies. The study examined the medical records of 1724 CLP subjects, distributed in different phases of dentition, assessing the presence of dental anomalies in association to the single dental element, to the type of cleft, to the gender, and to the location of the anomaly in relation to the skeletal area of the cleft. In relation to hypodontia of the maxillary arch, the study found that the most affected dental elements were the lateral incisors, both primary and permanent, and the second permanent premolar. The overall prevalence of hypodontia was 16.2% in primary dentition and 52.7% in permanent dentition. In both cases, the prevalence increased with the severity and extension of the defect and therefore of the cleft. Furthermore, in primary dentition, the prevalence was higher on the cleft side than on the contralateral side. In both dentitions, there were no differences between males and females and between the right and left sides. Microdontia mainly affected the permanent maxillary lateral incisors. Dental fusions were more frequent in primary than in permanent dentition and the main affected teeth were the primary upper central and lateral incisors, the corresponding lower central and lateral incisors, and the primary lower canines. The prevalence of supernumeraries was 17.7% in primary dentition and 5.7% in permanent dentition. The maxillary lateral incisors were mainly affected [13].

The association between CLP and enamel hypoplasia has been investigated for many years [15,16]. In a study for his 1961 Master’s thesis [16], Mink studied enamel hypoplasia in a sample of 98 CLP patients and reported a prevalence of 66.6% in primary dentition and 92.8% in permanent dentition. He also showed that the severity of the cleft (severity and extension of the defect) affected the severity of the enamel defect and its prevalence. A recent Chinese cross-sectional study [17] studied the association between CLP and various enamel defects, including demarcated opacity, diffuse opacity, and hypoplasia. The sample included 239 CLP patients and 469 non-CLP subjects with ages between 9 and 34 years. The overall prevalence of defects was significantly larger (*p* < 0.001) in subjects with CLP (87.9%) than in the control group (41.4%). The same disparity in prevalence was present for each single enamel defect, while no statistically significant difference between males and females was found.

A systematic review in 2019 [18] conducted on scientific articles of the last 20 years examined oral health problems related to the prevalence of caries and to oral hygiene patterns in patients with CLP. The 39 selected articles exhibited a high heterogeneity of type of study, observed population, evaluation periods, and considered variables. Nevertheless, the review notes a general consensus that pediatric patients with CLP tend to have higher plaque indices, higher prevalence of dental caries, and worse oral hygiene patterns than non-CLP subjects, hence the need to identify standardized protocols and to develop specific devices for the prevention and control of oral hygiene in the cleft area.

A case-control study of 2017 [19] examined dental caries and periodontal status of children and adolescents with CLP compared to a homogeneous control sample. The CLP sample included 156 patients between 5 and 18 years. The study measured DMFT and dmfs indexes, plaque index, gingival bleeding index, periodontal attachment level, and depth of the periodontal pocket and used these parameters to diagnose gingivitis and periodontitis. The study showed that the scores of all indices were significantly higher in the affected subjects than in the control sample and 29% of the subjects with CLP had generalized gingivitis compared to 1% of the unaffected subjects.

### 1.2. Multidisciplinary Approach in the Management of CLP Patients

Cleft lip and palate children benefit from a multidisciplinary team approach and special treatment requirements. Dental caries can be a crucial additional problem to these children: a good dietary awareness in relation to dental caries should be encouraged from early life, commencing with discussions between the mother and dental surgeon shortly after the birth of the child [20]. Prevention through sealants and varnishes should be performed when possible and individuals undergoing surgery should have an excellent oral condition, removing the sources of infection that may compromise the surgery. Supernumerary and/or malpositioned deciduous teeth adjacent to the cleft should be maintained as long as possible, in order to preserve bone tissue that is already defective in this region. 

In all the above-mentioned cases, the pediatric dentist plays a crucial role in achieving all these goals. Furthermore, the goal of the pediatric dentist within the CLP multidisciplinary team is to maximize oral motor function in affected children. This includes: working with the nutritionist to facilitate feeding; fabricating speech appliances as an aid to speech therapy; stabilizing and improving oral morphology to optimize surgical results and provide for optima masticatory function and esthetics. To accomplish these goals, three periods represent the different steps and evolutions to follow: newborn period, period of primary and mixed dentitions, and teenage period [21].

The value of a multidisciplinary team is widely known and mentioned in the literature, but very few papers focus on the role and the importance of the pediatric dentist. Therefore, the purpose of this article is to underline the role of a pediatric dentist as a member of the cleft lip and palate team which ranges from prenatal counseling, presurgical prevention and orthopedics to post-treatment rehabilitation and restoration. 

## 2. Materials and Methods

Literature searches of free text and MeSH terms were performed using the databases MedLine (PubMed) and Scopus from 2010 to July 2020. Double results from the databases were considered only once. All searches were conducted using a combination of subject headings and free-text terms. The final search strategy was determined through several pre-searches. The keywords and string of research used in the search strategy were as follows: (Cleft Lip AND pediatric dentist) OR (Cleft Palate AND pediatric dentist).

The articles that resulted from the research were 118 in total, but few of them included valuable notions and results related to the aim of this work. The ones mentioning the keywords, but not deepening the topic related to pediatric dentists, were excluded in the first screening. In the 11 remaining papers selected, there were no common factors that were sufficient to perform a metanalysis. Therefore, the authors chose to proceed in summarizing the key points in the result and discussion paragraph. 

## 3. Results and Discussion

### 3.1. Multidisciplinary Approach to Oral Health Problems in CLP and the Role of the Specialist in Pediatric Dentistry

If we examine the literature related to a multidisciplinary approach to CLP in the pediatric age, a case-control study of 2018 [22] assessed the oral health-related quality of life of CLP children and their families using the Early Childhood Oral Health Impact Scale (ECOHIS) questionnaire. The case group included children with surgically treated CLP while the control group consisted of children without CLP. The study showed that the overall quality of life score was different in the two groups. For the child, one of the reported problems was the difficulty of phonation, as the onset of the cleft during the development of motor control of the word could negatively impact phonation learning. Aesthetics was another largely reported problem. The study highlights how the implementation and maintenance of multidisciplinary intervention strategies are therefore required to restore facial aesthetics and oral functions and, above all, to improve the psychological support not only to CLP children but also to their families. A similar study, already mentioning these concepts and results, was carried out a year earlier by Rando et al., assessing oral health-related quality of life of children with CLP [23].

In light of these considerations, we must therefore underline how—as reported in several studies [24,25,26] a coordinated team of specialists is required for the management of the child affected by CLP, and within this team, the figure of the specialist in pediatric dentistry has a role of primary importance in the phase of clinical evaluation and treatment planning.

In the context of the therapeutic objectives of pediatric dentistry, the gold standard will be that of achieving and maintaining a good state of oral and dental health in the clinical management of all the described problems.

Ganesh et al. [24], in their works, thoroughly described the role of the pediatric dentist both in the pre-operative phase and in the post-operative one; these are summarized in Table 1. 

The outpatient preventive strategies are all based on the preliminary risk assessment of gingivitis, tooth decay and hypoplasia of the enamel [19,25]. By examining a triage of the oral prevention protocol, in low-risk conditions only professional oral hygiene sessions will be provided; in medium risk conditions topical applications of fluorine and sealing of permanent molars will be added; when the risk is high, minimally invasive conservative interventions are recommended, such as preventive resin restoration, or, when needed, more complex conservative restoration therapies.

### 3.2. Pediatric Dentistry Specialist Management of Dental Anomalies in Children with CLP

The clinical management approach to enamel hypoplasia in the child affected by CLP is substantially superimposable to that which is carried out in unaffected subjects. It is mainly based on the control of the diet, which must be low in carbohydrates, on motivating parents to apply home oral hygiene practices in the child’s early childhood, and on providing adequate oral hygiene instructions. Topical fluor prophylaxis and sealing of the furrows of the permanent molars will be provided and, finally, it will be necessary to schedule periodic follow-up checks on the state of dental health.

**Figure 1:**
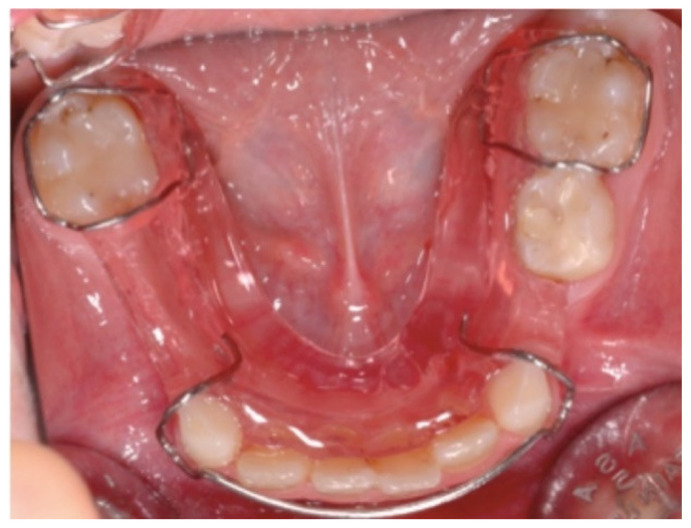
Example of orthodontic device aimed at maintaining space in the dental arch.

The therapeutic approach to hypodontia is primarily based on a preventive approach which includes the early initiation to oral hygiene maneuvers, the planning of professional oral hygiene sessions at regular intervals, and topical fluor prophylaxis. The main objective is, therefore, the preservation in the arch of the intact deciduous dental element and the maintenance of the dental arch length with the management of the arched space through the use of orthodontic devices with the aim of preventing the mesial migration of the adjacent dental elements (Figure 1). 

**Figure 2:**
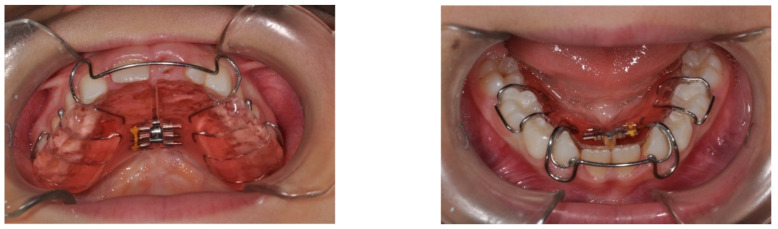
Examples of orthodontic plates with transversal screw.

Proper prevention in the clinical management of hypodontia must therefore consider the evaluation of the growth control of the dentoalveolar arches, which will therefore require the use of removable orthodontic plates equipped with auxiliary elements such as sagittal and transverse screws for growth control (Figure 2).

**Figure 3:**
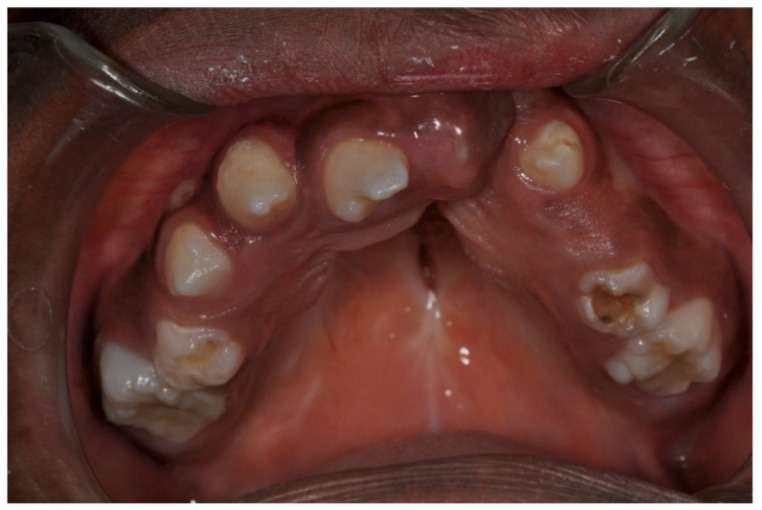
Example of patient with shape anomalies.

The clinical management of hypodontia in individuals with CLP must also be based on the evaluation of the potential presence of associated dental anomalies. In fact, especially in birth defects such as CLP, the prevalence of association between defective number anomalies (hypodontia) and shape anomalies is high (Figure 3). 

**Figure 4:**
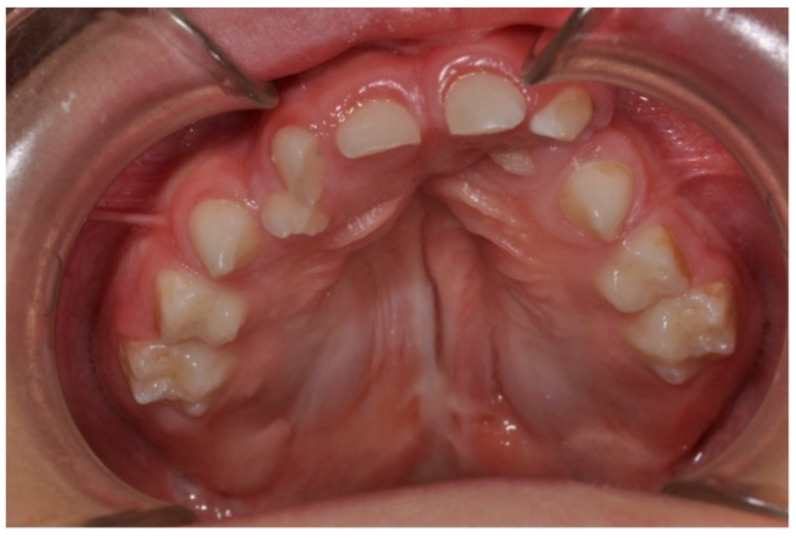
Delayed exfoliation of the deciduous elements and eruption delays.

Other associated anomalies may include the delayed exfoliation of the deciduous elements (Figure 4) and eruption delays. 

**Figure 5:**
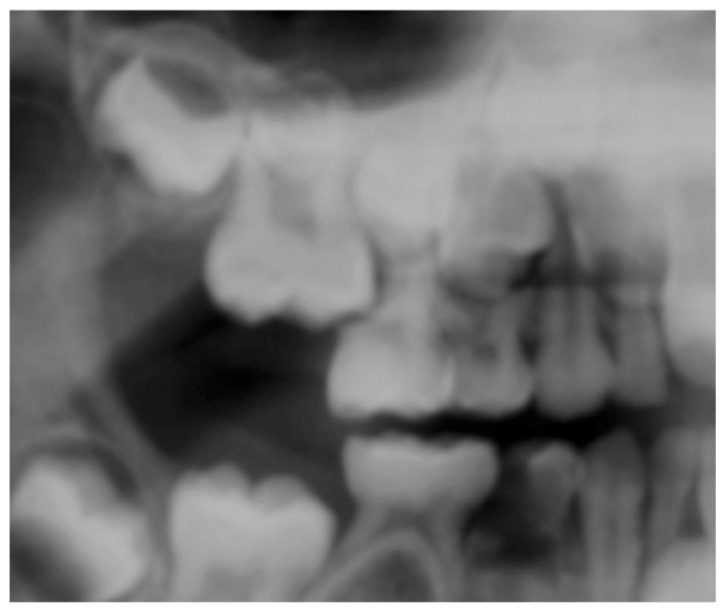
Example of impacted first permanent molar.

Therefore, the approach to hypodontia is complex as the pediatric dentist—together with the orthodontist—must not only manage the conservation of the deciduous elements and plan the preservation of the spaces in the arch while managing the growing length of the arches, but also evaluate the punctual timing of the exchange and tooth eruption control while monitoring contextual potential ectopic eruptions of the first permanent molar (Figure 5) or deciduous teeth exfoliation delays.

**Figure 6:**
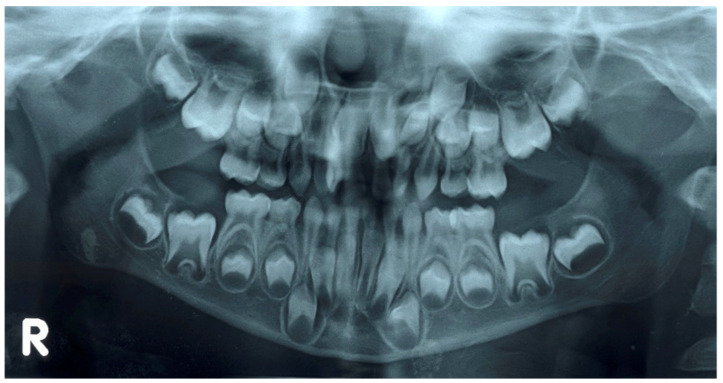
Example of Orthopantomography of patient with supernumerary tooth.

The clinical approach to the management of shape anomalies is based on the aesthetic rehabilitation of the dental elements affected by the morphological alteration, always associated with a preventive approach consisting in the search for concomitant anomalies, whose risk of appearance is greater in the late epoch, with the need, therefore, of scheduling timely controls of the dental exchange. The approach to polyodontia (Figure 6) always involves the use of first-level (e.g., Orthopantomography) and second-level (e.g., CBCT) radiographic examinations to evaluate all parameters which are related to the presence of supernumeraries in the pediatric age. 

These include the evaluation of the position of the supernumerary, its degree of formation, the degree of development of the contiguous dental elements, and the possible association of eruptive obstacles. Beyond these clinical factors, which are normally considered also in subjects without CLP, the correct approach to the clinical management of supernumeraries in pediatric patients with CLP imposes the postponement of the extraction surgery of the supernumerary, in order to prevent the operative traumatism from interfering with the dentoalveolar development of the affected area. In addition, if a combined orthodontic–surgical treatment is required, there is always the need to evaluate the growth phase of the subject. Finally, the clinical approach to dental inclusions in children with CLP will always aim at preventing the onset of mechanical, nervous, inflammatory, and dysplastic complications through an orthodontic treatment aimed at the recovery of space in the arch using removable devices with sectoral screws.

Furthermore, in relation to orthodontic problems associated with the period of deciduous and early mixed dentition in children with CLP, the literature reports a greater prevalence of mono dental crossbites linked to the anomalous position of the dental elements, as well as lateral posterior and anterior crossbites. Furthermore, the crossbite prevalence at 5 years is higher in children with cleft lip and palate than in children with cleft palate alone [27].

It is important, once again, to remark what was reported by Udin et al. [21], regarding the close collaboration between the pediatric dentist and the nutritionist in order to facilitate feeding; fabricating speech appliances as an aid to speech therapy; and stabilizing and improving oral morphology to optimize surgical results and provide for optima masticatory function and aesthetics.

Muhamad et al. [28], when describing how pediatric dentistry plays a critical role in creating a proper plan of care for oral health and overall nutrition, summarized and outlined other tasks related to the role of this figure in the team. As members of the cleft palate team, they help maintain healthy dentition and gums, monitor craniofacial growth and development, and correct jaw relationships and dental occlusion to achieve proper function and appearance. Feeding appliances and presurgical infant orthopedics appliance impressions are most frequently provided by the pediatric dentist on cleft palate teams at most hospital-based programs.

In dental rehabilitation, pediatric dentistry provides oral health information and should be able to follow the child with cleft lip and palate of the mixed dentition, craniofacial growth, and dentition development. The orthodontist monitors the craniofacial growth and development and corrects the malocclusions, which are more complex compared to patients without clefts.

Dogan et al., in their study regarding anxiety in the CLP patient, make an interesting note on how the pediatric dentist faces CLP children from very early childhood, and he/she is the responsible person who follows the patient in every stage. Furthermore, the pediatric dentist organizes the dental treatment plan and is usually responsible for assessing the dental anxiety status for these children and reporting it to their colleagues and instructing them about it [29].

Finally, there are surveys [30,31], that remark a positive parental attitude towards the provision of pediatric dental services—both dietary and oral status check-up.

## 4. Conclusions

The management of CLP patients in pediatric dentistry must always be based on the choice of therapeutic solutions related to the level of severity of the risk and on the recognition of the determining role of the compliance of the little patient.

The role of the pediatric dentist in pediatric dental care for the patient with a cleft lip and palate extends from birth through to adolescence and young adulthood and also plays a vital role in CLP multispecialty team. Apart from this, the pediatric dentist can also play a crucial role in communicating with the rest of the team. For the parents of babies with a cleft, the stages of cleft surgery are major landmarks: the dentist needs to understand the surgical procedures and their timing so that dental care can be integrated sensitively within the overall treatment plan. Two-way communication helps to achieve the most effective treatment plan for the individual. 

## Figures and Tables

**Table 1:** Role of the pediatric dentist.

Pre-Operative Role	Post-Operative Role
Parental counselling for diet and oral hygiene maintenance	Post-operative oral hygiene maintenance through professional oral hygiene maintenance aids
Early preventive advise for the child for caries prevention	Preventive care: topical fluoride and sealants application
Construction of feeding plate	Restorative care for the carious teeth and endodontic treatment for involved teeth
Presurgical orthopedics for correction or rotated premaxilla	Orthodontic correction of misaligned teeth
Instils positive attitude towards the dental treatment in a child by behaviour shaping or modification as required	Palatal plate for correction of speech problems

## Data Availability

Not applicable.
